# Endoscopic and combined approaches in the surgical management of frontal and ethmoidal sinus osteomas: a novel decision-making flowchart

**DOI:** 10.1007/s00405-026-10513-4

**Published:** 2026-08-03

**Authors:** Antonio Romano, Stefania Troise, Maria Esposito, Raffaele Spinelli, Eutilia Manzo, Vincenzo Abbate, Giovanni Dell’Aversana Orabona

**Affiliations:** Maxillofacial Surgery Unit, Department of Neurosciences, Reproductive and Odontostomatological Science, University of Naples Federico II, Via Sergio Pansini 5, Naples, 80138 Italy

**Keywords:** Paranasal sinus osteoma, Endoscopic surgery, Combined approach, Intraoperative navigation, Patient-specific guide, Surgical decision-making

## Abstract

**Purpose:**

Paranasal sinus osteomas are benign but potentially symptomatic lesions whose management remains heterogeneous despite recent advances. Choosing between a purely endoscopic or a combined approach can be challenging, particularly for larger lesions. This study aims to present our institutional experience in the surgical management of paranasal sinus osteomas and to propose a flowchart that integrates clinical and radiological findings and modern technologies, including intraoperative navigation and patient-specific cutting guides, to assist clinicians in selecting the optimal surgical approach.

**Methods:**

A retrospective review was conducted on patients who underwent surgical resection of frontal and ethmoidal sinus osteomas at the Maxillofacial Surgery Unit of the University of Naples “Federico II” between January 2020 and April 2024. Patients were classified according to anatomical grading and surgical approaches were categorized as endoscopic or combined. Demographic data, radiological features, surgical details, and postoperative outcomes were analyzed. Based on these findings, our flowchart was developed.

**Results:**

22 patients (13 males, 9 females; mean age = 51.5 years) were included. 9 (41%) were Type I, 7 (32%) Type II, and 6 (27%) Type III osteomas. Endoscopic surgery was performed in 16 cases (73%), while combined approaches were required in 6 cases (27%), exclusively for Type III lesions. No major postoperative complications or recurrences were observed during a mean follow-up of 40.3 months (20–68 months).

**Conclusions:**

Endoscopic treatment is effective for most fronto-ethmoidal osteomas, while combined approaches remain indicated for large or extended lesions. This flowchart offers a guide for selecting endoscopic or combined approaches, enhancing safety and outcomes.

## Introduction

Paranasal sinus osteomas are benign, slow-growing osseous tumors arising from the walls of the paranasal sinuses. Despite being among the most common benign craniofacial bone lesions, their pathogenesis and optimal management remain debated [1, 2]. Imaging-based studies report a prevalence ranging from 0.01% to 3% in the general population [3]. The frontal sinus is most frequently involved, followed by the ethmoid sinus, whereas maxillary and sphenoid localizations are rare [4]. Most osteomas are incidentally detected and remain asymptomatic. However, progressive growth, sinus outflow obstruction, or extension toward the orbit or anterior cranial fossa may result in symptoms such as headache, facial pain, nasal obstruction, or orbital complications, and rarely intracranial involvement [5]. Computed tomography is the imaging modality of choice, as it accurately defines lesion size, density, and its relationship with the orbit and skull base, guiding surgical planning [6]. Asymptomatic and stable osteomas are generally managed conservatively with radiological surveillance, whereas surgery is recommended for symptomatic, enlarging, or extensive lesions, particularly when sinus drainage is compromised or orbital or intracranial extension is present [7]. Traditionally, external osteoplastic flap procedures represented the standard surgical approach, offering wide exposure of the frontal sinus but at the cost of higher morbidity and aesthetic impact [8]. With the advent of endoscopic sinus surgery, management has progressively shifted toward minimally invasive techniques, allowing effective resection with reduced morbidity and faster recovery [9]. Nonetheless, lesion size, lateral extension, or posterior table involvement may limit a purely endoscopic approach, making combined or open procedures necessary in selected cases. Several classification systems have been proposed to guide the selection of the surgical approach. The grading model of Chiu stratified frontal sinus osteomas based on size and relation to the lamina papyracea, suggesting that lesions medial to this plane were suitable for endoscopic removal, while lateral or superiorly attached tumors required open access [10]. More recently, the contributions of Castelnuovo and Prizio have led to a refinement of both anatomical classifications and surgical indications for frontal and fronto-ethmoidal osteomas, reflecting the impact of modern endoscopic techniques, navigation systems and extended frontal sinus approaches [11, 12]. According to this anatomical model, Grade I osteomas are small (≤ 3 cm) and confined medially to the lamina papyracea, and are therefore generally suitable for a purely endoscopic approach. Grade II lesions, also ≤ 3 cm, extend laterally up to the midpoint of the orbital roof and typically require extended endoscopic techniques. Conversely, Grade III osteomas are larger than 3 cm or extend laterally to the mid-orbital plane, often involving the lateral frontal sinus, and have traditionally been managed through combined or open approaches, sometimes with frontal sinus obliteration.

Despite these efforts, a standardized algorithm that combines clinical, radiological, and technological parameters for decision-making is still lacking. The present study aims to contribute to this evolving field by reporting a single-institution experience in the surgical management of frontal and ethmoidal sinus osteomas. Based on the integration of clinical findings, radiologic assessment, and modern technological tools, this study proposes a novel decision-making flowchart designed to optimize surgical planning and the choice between endoscopic or combined approaches. This model seeks to provide a practical and reproducible reference for clinicians dealing with paranasal sinus osteomas, ensuring a balance between surgical radicality and preservation of anatomical and functional integrity.

## Materials and methods

### Study design and ethical approval

This study was designed as a retrospective single-center chart review of patients who underwent surgical resection of paranasal sinus osteomas at the Maxillofacial Surgery Unit of the University of Naples “Federico II,” between January 2020 and April 2024. Ethical review and approval were obtained from the Ethics Committee of the University of Naples “Federico II” (Protocol No. 32/2024). The study was conducted in accordance with the principles of the Declaration of Helsinki. Written informed consent was obtained from all patients before inclusion in the study.

### Patient selection

All patients who underwent surgery for paranasal sinus osteomas during the study period were retrospectively analyzed.

Patients who satisfied the following inclusion criteria were enrolled in the study:

- Diagnosis of paranasal sinus osteoma, radiologically confirmed by CT performed within 7 days before the surgery;
- Surgical treatment performed using either an endoscopic, open, or combined approach;
- A minimum follow-up of 18 months.

The exclusion criteria were:

- Patients managed conservatively with periodic follow-up outpatients checks;
- Patients with a history of facial trauma or chemo-radiotherapy affecting the frontal region;
- Patients refusing surgery;
- Cases with incomplete imaging or follow-up data.

The requirement for CT acquisition within 7 days before surgery was adopted to ensure consistency between preoperative imaging and intraoperative anatomy. This interval was particularly relevant in cases requiring patient-specific cutting guides, as it minimized potential discrepancies between the segmented radiological dataset and the anatomical surface used for guide fitting.

A total of 22 patients satisfying these criteria were included in the analysis.

### Data collection

Data collection included demographic variables, clinical presentation, tumor size and location, anatomical relationships, and the type of surgical approach. Preoperative sinonasal symptoms and their impact on quality of life were assessed using the Sinonasal Outcome Test-22 (SNOT-22), a validated patient-reported questionnaire comprising 22 items related to sinonasal symptoms, sleep quality, functional limitations, and emotional well-being. Each item is rated on a six-point Likert scale ranging from 0 (*no problem*) to 5 (*problem as bad as it can be*), resulting in a total score ranging from 0 to 110. Higher scores indicate a greater symptom burden and a more pronounced impairment of health-related quality of life. High-resolution computed tomography (CT) scans of the paranasal sinuses were obtained for all patients and used to assess the size, shape, and topographic relations of each lesion with adjacent structures such as the orbital roof and anterior cranial fossa. Radiological analysis was essential for accurate surgical planning and the development of a personalized management strategy.

**Fig. 1:**
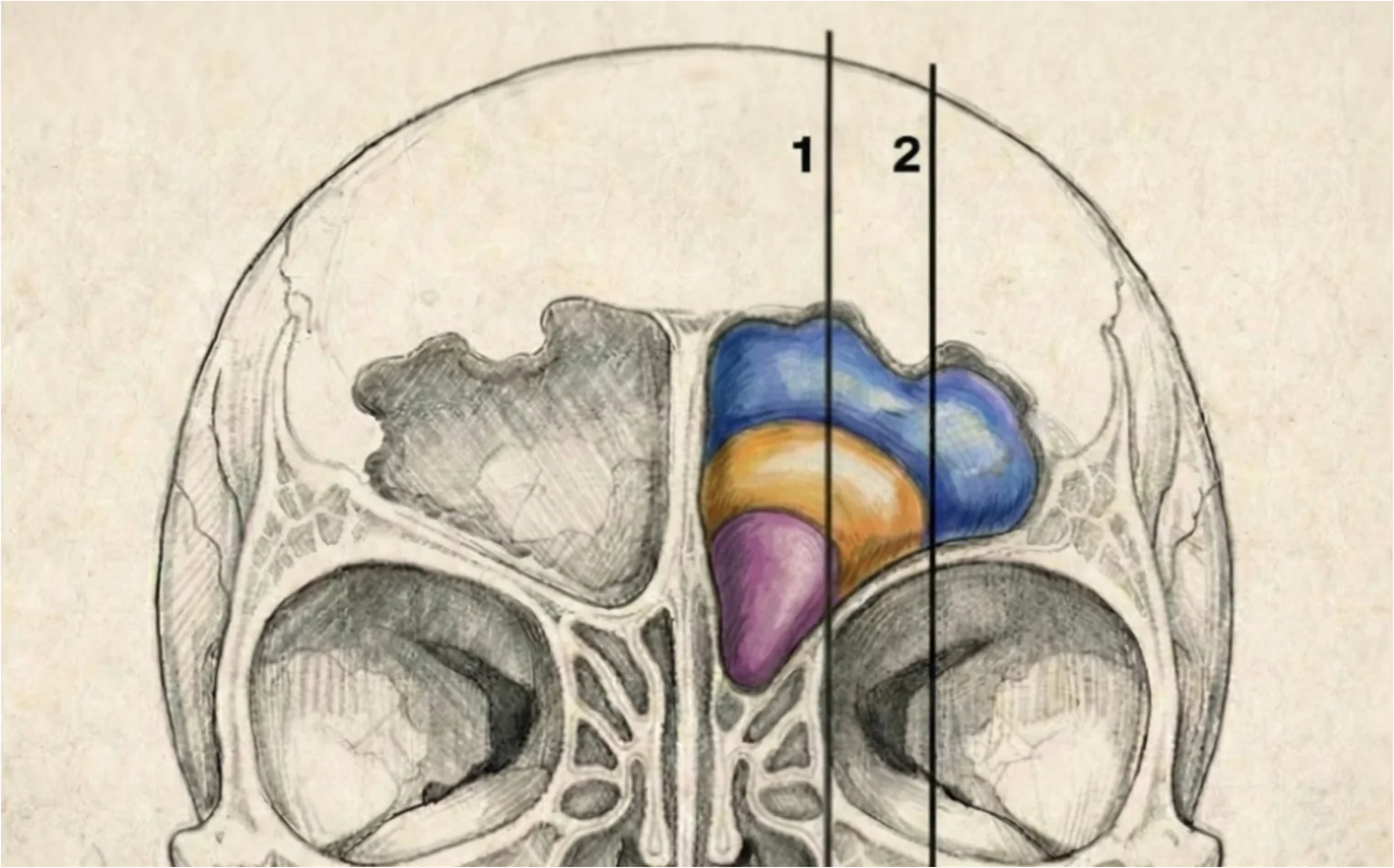
Schematic representation of osteomas occupying the frontal sinus. Two sagittal reference planes, passing through the lamina papyracea and the midpoint of the orbital roof, are shown to define lesion extension

Based on coronal CT imaging, frontal sinus osteomas were evaluated using an integrated anatomical framework derived from Prizio’s classification and refined according to the endoscopic experience of Castelnuovo and colleagues [11, 12]. This model divides the frontal sinus by two sagittal planes, through the lamina papyracea and the midpoint of the orbital roof, and incorporates lesion size, lateral extension, site of attachment, and frontal sinus table involvement into a three-type classification system. (Fig. 1).

According to these classifications, the osteomas can be divided in three types:

- Type I osteomas are lesions measuring ≤ 3 cm, confined medially to the lamina papyracea, with an inferior or posterior–inferior attachment accessible through the frontal recess. In this group, frontal sinus table involvement is absent or limited to minimal posterior table contact without erosion.
- Type II osteomas include lesions ≤ 3 cm extending laterally up to the midpoint of the orbital roof. These lesions typically show a posterior or superior attachment that remains reachable with angled endoscopic visualization and may present limited posterior table contact without erosion.
- Type III osteomas are characterized by a size > 3 cm or extension lateral to the mid-orbital plane into the lateral frontal sinus. They usually present a broad-based, superior or lateral attachment and frequently show anterior and/or posterior table involvement, with possible bony erosion.

In addition to lesion size and lateral extension, particular attention was paid to anatomical variables that could directly influence the surgical strategy, including the site of osteoma attachment, involvement of the anterior or posterior frontal sinus tables, accessibility of the frontal recess, relationship with the orbit and skull base, and the feasibility of restoring physiological frontal sinus drainage. These parameters were systematically evaluated during preoperative planning and subsequently incorporated into the proposed decision-making algorithm.

### Flowchart development

The proposed decision-making flowchart was developed through a retrospective evaluation of the surgical management strategies adopted in all patients included in the present series. While the anatomical classification of osteomas represented the initial framework for treatment selection, additional variables that consistently influenced surgical planning were identified and integrated into the algorithm. These included the site of lesion attachment, involvement of the anterior or posterior frontal sinus tables, accessibility of the frontal recess, relationships with the orbit and skull base, feasibility of restoring physiological frontal sinus drainage, and the availability of adjunctive technologies such as intraoperative navigation and patient-specific cutting guides.

The final algorithm was generated by correlating preoperative radiological findings, intraoperative anatomical observations, and the surgical approach ultimately selected in each case. Unlike previously published classification systems, which primarily rely on lesion size and lateral extension, the proposed flowchart incorporates additional anatomical and technological variables that directly influence modern surgical planning, thereby providing a more comprehensive framework for selecting the most appropriate surgical strategy.

### Cases presentation

Basing on these grading principles, this study reports three emblematic cases that reflect the spectrum of osteoma presentation and the tailored management strategies. All surgeries were performed by the same senior surgeon with over 25 years of experience in endoscopic and craniofacial surgery, ensuring procedural consistency and minimizing operator-dependent variability.

#### Case 1 – type I fronto-ethmoidal osteoma

The first case involved a 55-year-old woman presenting with intermittent frontal headache and mild nasal obstruction. Preoperative CT imaging revealed a small, well-circumscribed, pedunculated osteoma measuring less than 3 cm in maximum diameter, localized medially to the lamina papyracea and classified as a Type I fronto-ethmoidal osteoma. The lesion was completely removed using a purely endoscopic endonasal approach (Karl Storz GmbH & Co. KG, Tuttlingen, Germany). After induction of general anesthesia, a complete endoscopic endonasal approach was performed.

**Fig. 2:**
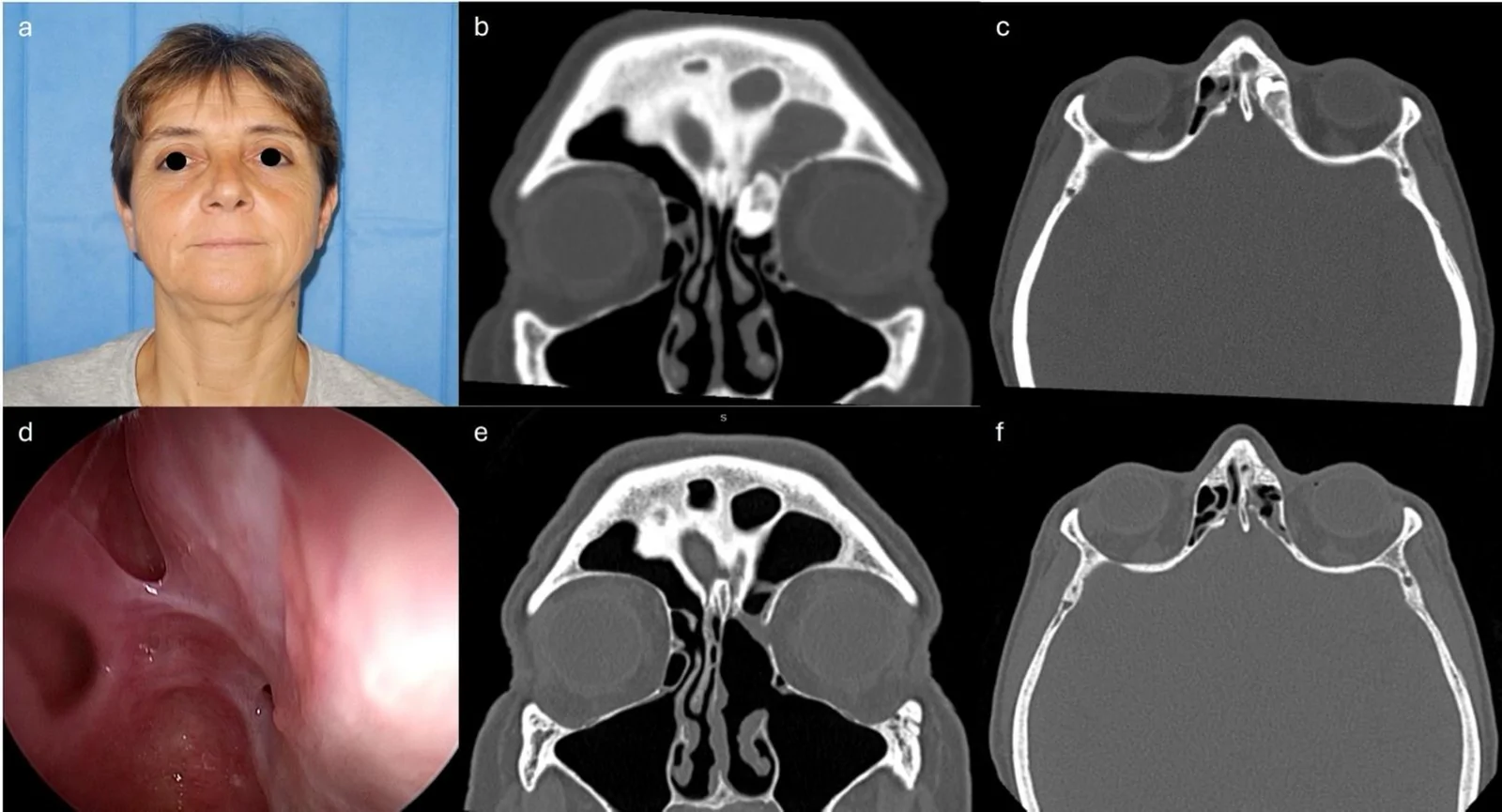
Case 1 – Type I Fronto-Ethmoidal Osteoma. (**a**) Preoperative frontal clinical photograph. (**b**–**c**) Preoperative coronal and axial CT scans showing the osteoma. (**d**) Postoperative endoscopic view after complete removal. (**e**–**f**) Postoperative coronal and axial CT scans confirming full excision

**Fig. 3:**
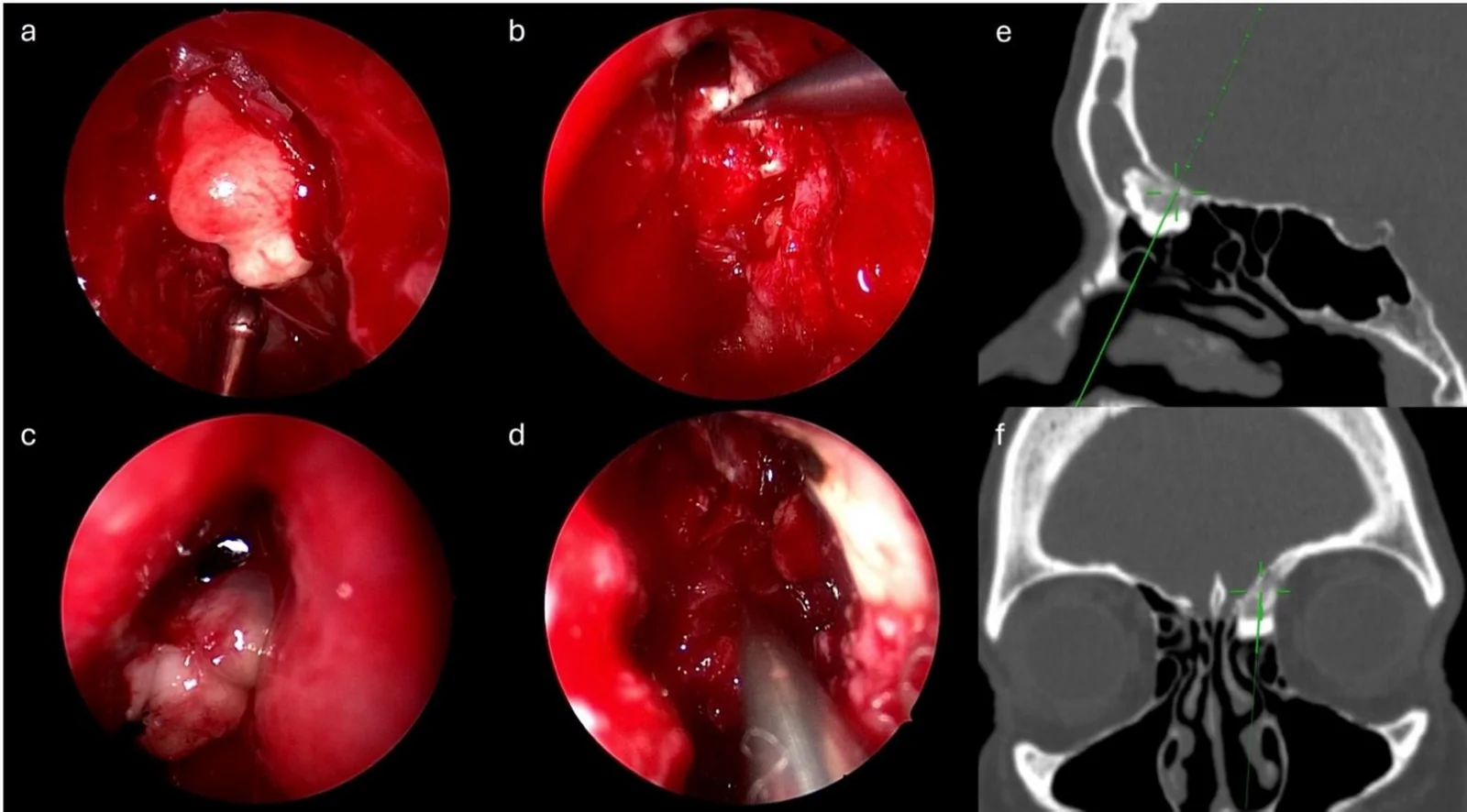
Intraoperative endoscopic frames of Case 1. (**a**) Exposure of the frontal recess osteoma. (**b**) Neuronavigation-assisted assessment of the residual osteoma. (**c**) Dislocation and mobilization of the cranial portion of the osteoma. (**d**) Final endoscopic view of neuronavigation check confirming complete removal; (**e**-**f**) Neuronavigation check of complete removal

A standard uncinectomy was performed, followed by an anterior ethmoidectomy with removal of the ethmoidal bulla and opening of the basal lamella to gain access to the frontal recess. A Draf IIa frontal sinusotomy was performed to ensure adequate access to the frontal recess. Under intraoperative navigation, the tumor was progressively thinned with a diamond burr and detached from its pedicle using angled instruments under endoscopic visualization (0°, 45°, 70°). The osteoma was then removed, and careful attention was paid to preserving the sinus mucosa and maintaining a wide neo-ostium to prevent postoperative stenosis. The skull base and orbital lamina were kept intact. The patient’s postoperative course was uneventful, and she was discharged on the third day with instructions for saline irrigations and topical corticosteroids. At 12-month follow-up, endoscopic and radiological evaluations confirmed complete healing, normal sinus ventilation, and no evidence of recurrence Figs. 2 and 3.

#### Case 2 – type II fronto-ethmoidal osteoma

The second case concerned a 62-year-old male patient with a five-year history of progressive nasal obstruction and recurrent frontal headaches. CT scans demonstrated a pedunculated lesion extending medially up to the mid-orbital line, consistent with a Type II fronto-ethmoidal osteoma. According to its size and location, the tumor was managed through an extended endoscopic approach.

**Fig. 4:**
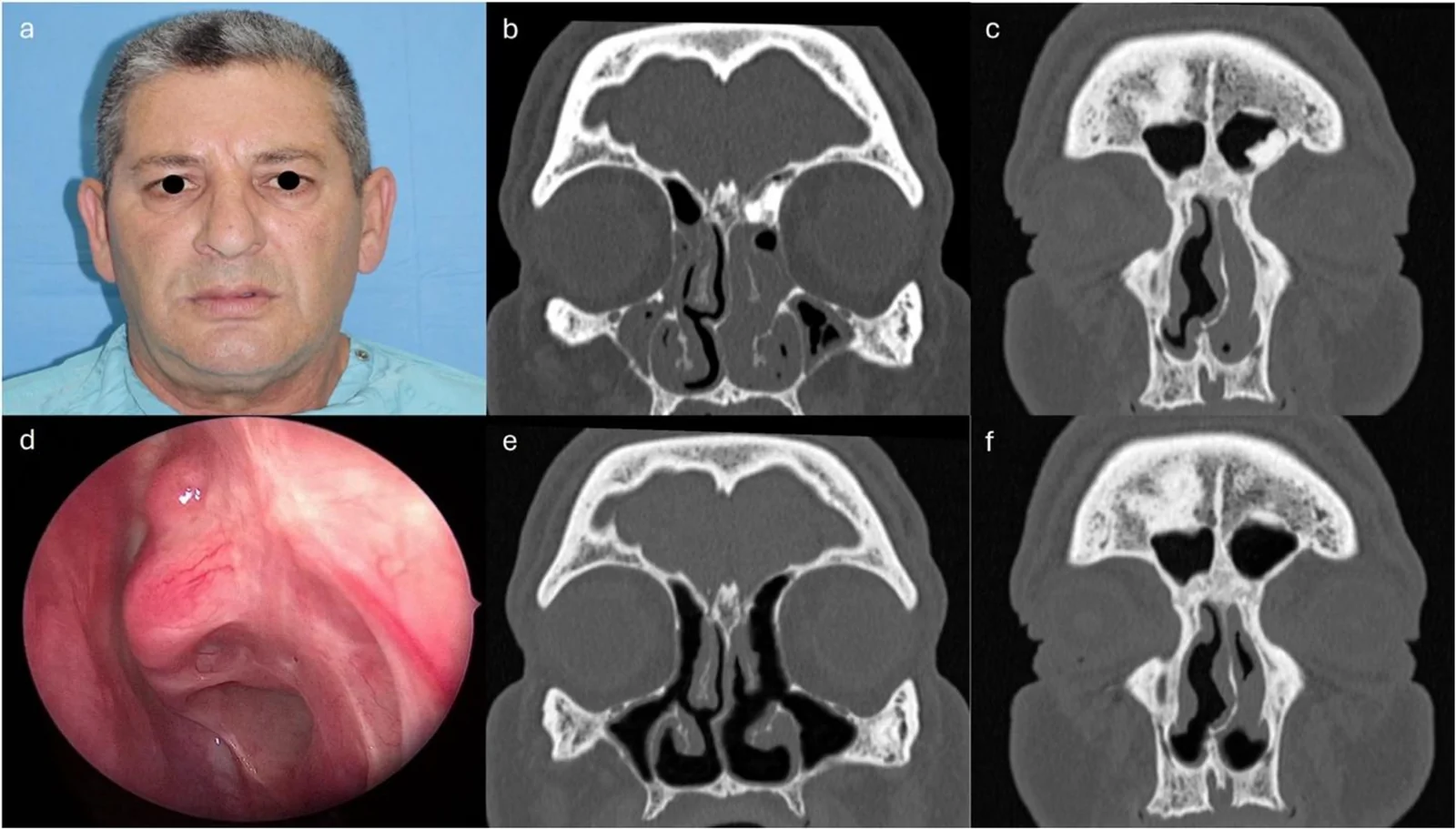
Case 2 – Type II Fronto-Ethmoidal Osteoma. (**a**) Preoperative frontal clinical photograph. (**b**–**c**) Preoperative coronal CT scans showing the osteoma at the frontal recess and anterior frontal sinus. (**d**) Postoperative endoscopic view after removal. (**e**–**f**) Postoperative coronal CT scans confirming complete excision and restoration of frontal sinus ventilation

**Fig. 5:**
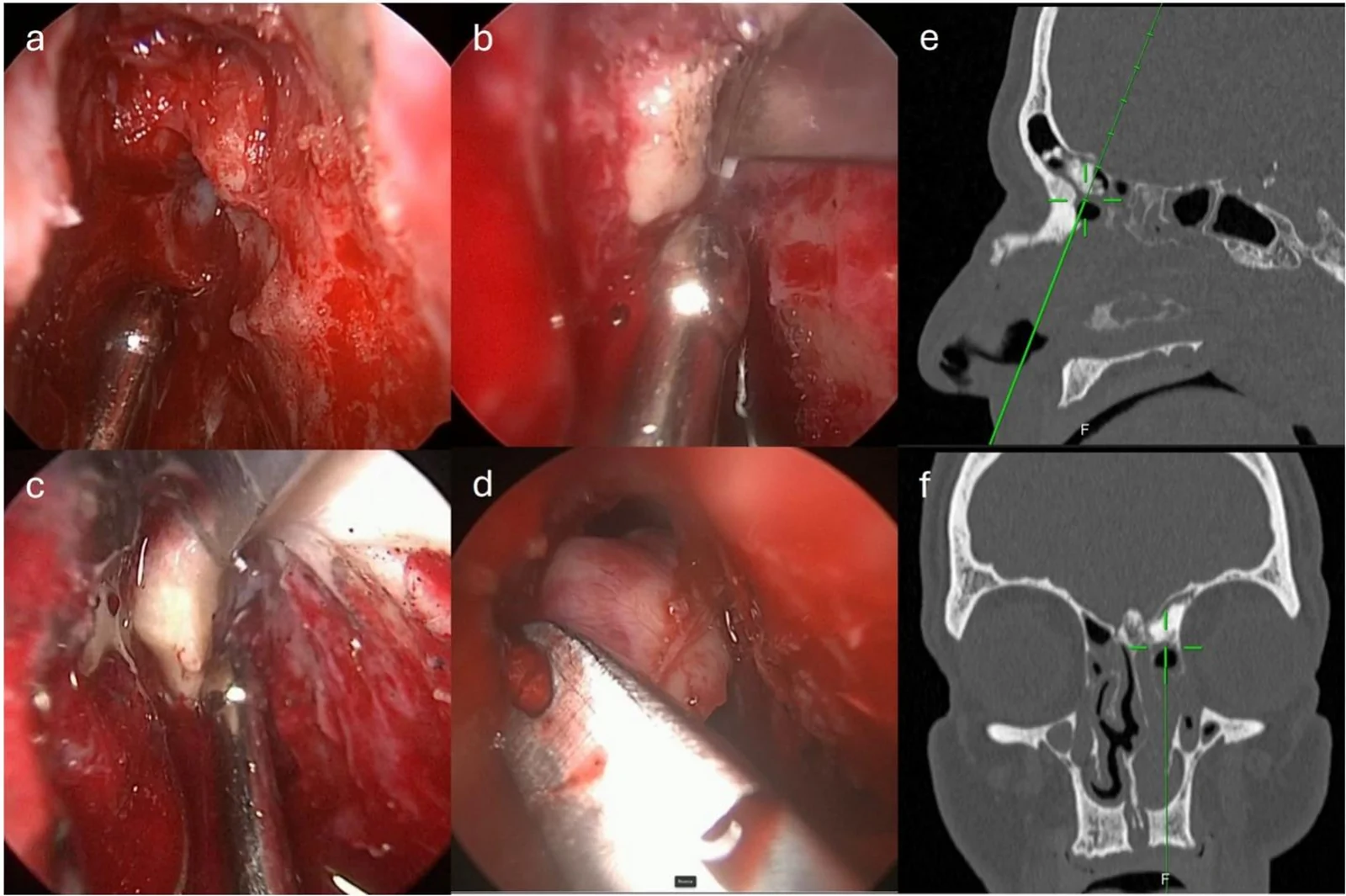
Intraoperative endoscopic frames of Case 2. (**a**) Exposure of the osteoma in the left frontal recess. (**b**) Piezoelectric segmentation of the lesion. (**c**) Mobilization and sectioning of the cranial portion. (**d**) Removal of the supraorbital component. (**e**–**f**) Neuronavigation control of osteoma extension in sagittal and coronal views

A standard uncinectomy was performed to access the anterior nasal cavity and maxillary ostium. This step was followed by an anterior and posterior ethmoidectomy, which progressively exposed the frontal recess [13]. Using high-definition angled endoscopes and powered instruments, osteoma was carefully skeletonized, sagittal osteotomies were performed to allow safe and precise removal of the bony segment, minimizing the risk of damage to adjacent structures. Then a Draf IIb frontal sinusotomy was performed under real-time image-guided navigation to manage the superior portion of the frontal sinus and ensure adequate visualization and ventilation of the frontal recess. The endoscopic field was continuously verified with navigation to minimize the risk of orbital or skull base injury. The postoperative period was uneventful, and at 12-month follow-up, both CT imaging and endoscopic inspection confirmed complete removal of the osteoma, restoration of frontal sinus ventilation, and absence of recurrence Figs. 4 and 5.

#### Case 3 – type III frontal sinus osteoma

The third case involved a 54-year-old man who presented with a six-year history of chronic frontal headache, left-sided epiphora, left-side globe lateralization, nasal congestion, progressive exophthalmos, and diplopia. CT revealed a large osteoma occupying the left frontal sinus, with lateral extension beyond the mid-orbital plane and partial erosion of the posterior table, consistent with a Type III frontal sinus osteoma.

Given the lesion’s size, lateral extension, and close relationship with the orbit and skull base, a combined endoscopic–external approach was planned to achieve complete resection while preserving both aesthetic and functional integrity. The procedure was performed involving two coordinated surgical teams operating simultaneously, one performing the endonasal endoscopic dissection and the other conducting the open coronal approach. The endoscopic team began by performing a uncinectomy, bilateral anterior and posterior ethmoidectomy, allowing visualization of the medial orbital wall and the ethmoidal portion of the osteoma. A Draf IIb frontal sinusotomy was then carried out to access the frontal recess. This endoscopic phase provided inferior and medial exposure of the lesion, enabling safe guidance and control for the concurrent external dissection. Simultaneously, the external team performed a coronal incision and raised a galea flap. The frontal bone was exposed, and a patient-specific 3D-printed cutting guide, designed preoperatively from CT segmentation, was positioned on the anterior wall until a perfect fit was achieved and secured with titanium screws. The use of the 3D cutting guide was carried out in accordance with the standardized protocol developed at the University of Naples “Federico II” [14, 15].

**Fig. 6:**
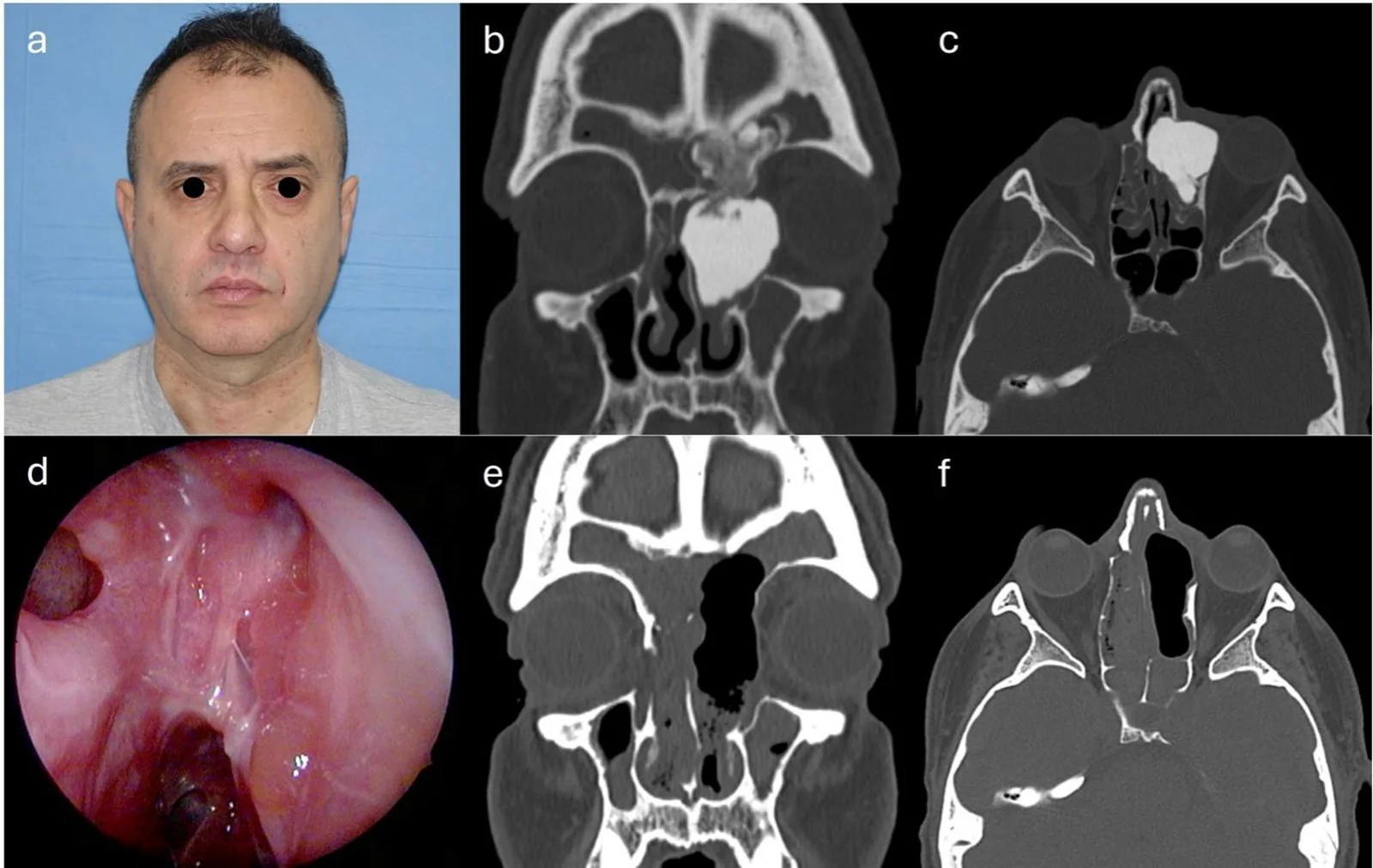
Case 3 – Type III Frontal sinus osteoma. (**a**) Preoperative frontal clinical photograph. (**b**–**c**) Preoperative coronal and axial CT scans showing the extent of the lesion. (**d**) Postoperative endoscopic view following removal. (**e**–**f**) Postoperative coronal and axial CT scans confirming complete excision

**Fig. 7:**
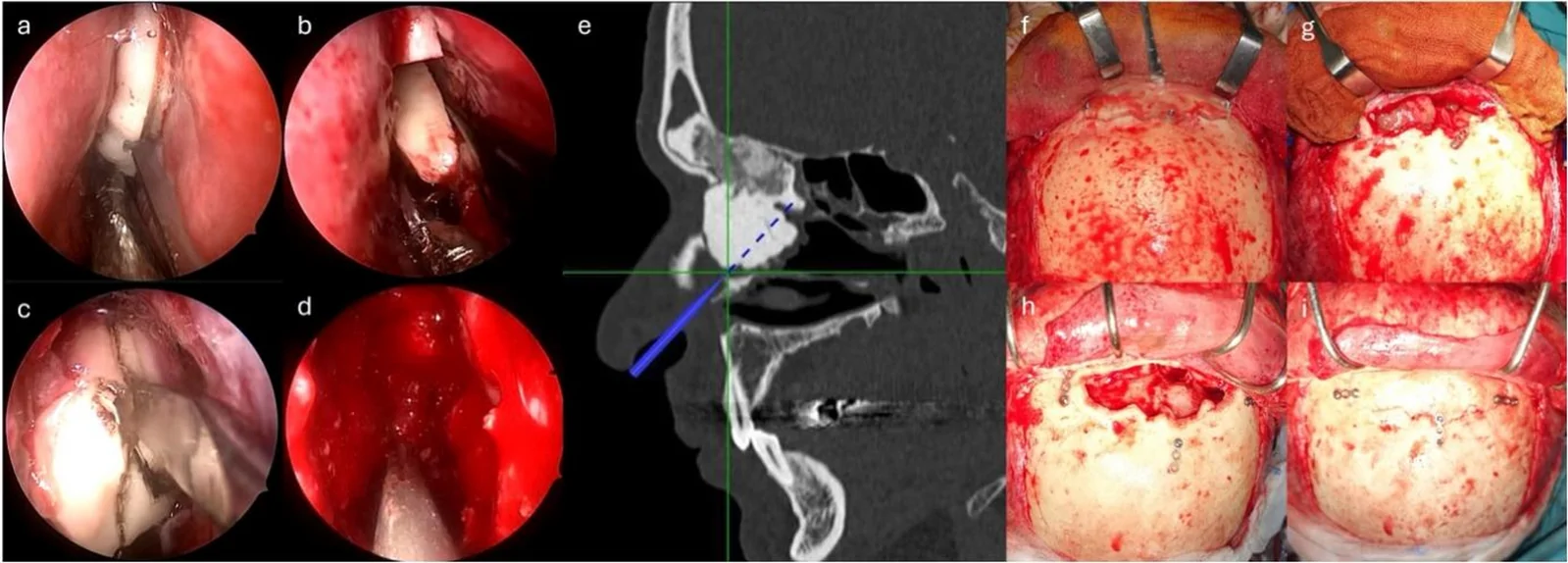
Intraoperative frames of Case 3. (**a**) Endoscopic Exposure and piezoelectric segmentation of the osteoma in the left middle meatus. (**b**) Removal of the middle meatal component of the osteoma. (**c**) Piezoelectric segmentation of the anterior ethmoidal portion. (**d**) Intraoperative neuronavigation check of the osteoma. (**e**) Neuronavigation check in sagittal view. (**f**) External view and custom-made cutting guide positioning. (**g**) Opening of the frontal sinus and exposure of the intrasinusal portion of the osteoma. (**h**) Frontal sinus after complete removal. (**i**) Obliteration of the frontal sinus with abdominal fat graft and repositioning of the bone flap

Osteotomies were performed with piezosurgery at a 45° inclination along the guide margins, allowing the controlled detachment of the frontal bone bandeau and the subsequent repositioning of the bone flap. Once the frontal sinus was opened, the mass was segmented by using Piezosurgery and then drilled up to complete resection and visualization of healthy bone [16]. After the complete removal of the lesion, restoration of frontal sinus ventilation was not possible due to the extent of the osteoma and the anatomic distortion of the frontal outflow tract. Therefore, complete removal of the frontal sinus mucosa was performed, followed by sinus obliteration. After meticulous mucosal stripping, the nasofrontal duct was sealed, and the cavity was thoroughly drilled and smoothed to remove any residual mucosa or septations. Autologous abdominal fat was then harvested and packed within the neo-cavity to obliterate the sinus completely, ensuring long-term separation from the nasal cavity and preventing mucocele formation. Then the frontal bone bandeau was fixed with miniplates. The postoperative recovery was uneventful, and the patient was discharged on day 6. At the 18-month follow-up, both clinical and radiologic assessments confirmed the absence of recurrence or residual deformity, with complete resolution of diplopia, globe asymmetry and restoration of ocular motility Figs. 6 and 7.

### Follow-up and outcomes

All patients underwent regular outpatient follow-up visits at 1 week, 1 month, 6 months, 12 months postoperatively. At 12 months, all patients underwent a postoperative CT scan to evaluate the presence of recurrence. Subsequent follow-up evaluations were scheduled annually. Postoperative outcomes were assessed through clinical examination, nasal endoscopy, radiological imaging, and patient-reported sinonasal quality of life. The Sinonasal Outcome Test-22 (SNOT-22) questionnaire was administered during postoperative follow-up. Headache evolution was recorded clinically as complete resolution, partial improvement, or persistence. In patients in whom physiological frontal sinus drainage was preserved, endoscopic evaluation included assessment of frontal neo-ostium patency, categorized as patent, stenotic, or occluded. In patients who underwent frontal sinus obliteration, ostium patency assessment was not applicable, and follow-up focused on the detection of delayed complications, including mucocele formation. Since no validated cosmetic scale was used, aesthetic outcomes were reported descriptively on the basis of the presence or absence of clinically evident scars or forehead contour irregularities.

## Results

### Population features

Following inclusion criteria, a total of 22 patients were enrolled in the study. The general features of the sample are described in Table 1.

**Table 1:** Main features of the 22 enrolled patients

Case *N*°	Age (yrs)	Sex	Preoperative symptoms (SNOT-22 pre-op)	Type	Surgical approach	Surgical procedure time	Follow-up	Outcomes (SNOT-22 post-op)
1	44	M	Frontal headache (SNOT-22 = 18)	I	Endoscopic	128 min	68 months	Resolution of symptom (SNOT-22 = 4)
2	55	F	Frontal headache, mild facial pressure (SNOT-22 = 22)	I	Endoscopic	127 min	59 months	Resolution of symptoms (SNOT-22 = 5)
3	28	M	Frontal pain, nasal obstruction (SNOT-22 = 28)	I	Endoscopic	140 min	53 months	Resolution of symptoms (SNOT-22 = 6)
4	60	M	Headache, recurrent sinusitis (SNOT-22 = 35)	I	Endoscopic	190 min	47 months	Resolution of symptoms (SNOT-22 = 10)
5	49	F	Frontal pressure, Headache (SNOT-22 = 24)	I	Endoscopic	122 min	45 months	Resolution of symptoms (SNOT-22 = 5)
6	45	M	Frontal pain, nasal obstruction (SNOT-22 = 30)	I	Endoscopic	112 min	40 months	Resolution of symptoms (SNOT-22 = 7)
7	50	F	Headache (SNOT-22 = 16)	I	Endoscopic	135 min	33 months	Partial resolution of symptoms (SNOT-22 = 11)
8	59	F	Frontal pain, localized swelling (SNOT-22 = 20)	I	Endoscopic	119 min	26 months	Resolution of symptoms (SNOT-22 = 4)
9	41	M	Frontal pain, headache (SNOT-22 = 19)	I	Endoscopic	133 min	24 months	Resolution of symptoms (SNOT-22 = 5)
10	62	M	Headache, nasal obstruction (SNOT-22 = 32)	II	Endoscopic	131 min	63 months	Resolution of symptoms (SNOT-22 = 8)
11	54	F	Frontal pain, headache (SNOT-22 = 25)	II	Endoscopic	130 min	55 months	Resolution of symptoms (SNOT-22 = 6)
12	57	M	Headache, nasal obstruction (SNOT-22 = 34)	II	Endoscopic	128 min	50 months	Resolution of symptoms (SNOT-22 = 9)
13	48	F	Headache, nasal obstruction. (SNOT-22 = 27)	II	Endoscopic	90 min	47 months	Partial resolution of symptoms (SNOT-22 = 14)
14	50	M	Headache, nasal obstruction (SNOT-22 = 29)	II	Endoscopic	125 min	43 months	Resolution of symptoms (SNOT-22 = 7)
15	68	M	Frontal pain, Headache (SNOT-22 = 21)	II	Endoscopic	126 min	37 months	Resolution of symptoms (SNOT-22 = 5)
16	53	F	Headache (SNOT-22 = 17)	II	Endoscopic	144 min	29 months	Resolution of symptom (SNOT-22 = 4)
17	31	M	Headache, lateral globe dislocation (SNOT-22 = 31)	III	Combined + sinus obliteration	255 min 23 min	44 months	Partial resolution of symptoms (SNOT-22 = 18)
18	57	F	Headache, dystopia, exophthalmos, lateral globe dislocation (SNOT-22 = 38)	III	Combined + sinus obliteration	300 min 20 min	34 months	Partial resolution of symptoms (SNOT-22 = 20)
19	48	M	Asymptomatic (SNOT-22 = 6)	III	Combined + sinus obliteration	210 min 20 min	27 months	Asymptomatic (SNOT-22 = 5)
20	55	M	Frontal swelling, headache (SNOT-22 = 23)	III	Combined	180 min 24 min	22 months	Resolution of symptoms (SNOT-22 = 6)
21	63	F	Frontal swelling, nasal obstruction, headache (SNOT-22 = 33)	III	Combined + sinus obliteration	235 min 26 min	21 months	Resolution of symptoms (SNOT-22 = 8)
22	54	M	Frontal headache, epiphora, nasal obstruction, exophthalmo, and diplopia. (SNOT-22 = 41)	III	Combined	260 min 27 min	20 months	Resolution of symptoms (SNOT-22 = 9)

The sample included 13 males and 9 females; the mean age at surgery was 51.5 years (range 28–68 years). The frontal sinus was the most frequently affected site (15 cases, 68.2%), followed by the fronto-ethmoidal complex (5 cases, 22.7%) and the ethmoidal sinus alone (2 cases, 9.1%). No osteomas were located in the sphenoid or maxillary sinuses. Symptoms at presentation varied according to tumor localization. The most common manifestation was headache (16 patients, 72.7%), followed by nasal obstruction (8 patients, 36.4%), facial pressure or pain (6 patients, 27.3%), and orbital symptoms such as diplopia, epiphora, or exophthalmos (3 patients, 13.6%). Only one lesion (4.5%) was discovered incidentally during imaging performed for unrelated reasons.

A significant improvement in sinonasal symptom burden was observed during postoperative follow-up. The mean SNOT-22 score decreased from 25.9 ± 8.3 preoperatively to 8.0 ± 4.4 postoperatively, with a mean reduction of 17.9 ± 6.9 points. The median score decreased from 26 (range: 6–41) to 6.5 (range: 4–20). Overall, 20 of 22 patients showed a reduction of at least 9 points, consistent with a clinically relevant improvement in sinonasal quality of life.

According to the anatomical classification, 9 patients (41%) had Type I, 7 (32%) had Type II, and 6 (27%) had Type III osteomas.

Endoscopic approaches were performed in 16 cases (73%), while 6 patients (27%) required a combined endoscopic–external approach. No patient underwent a purely open procedure.

The mean operative time was 130 min (range 90–190 min) for endoscopic procedures and 240 min (range 180–300) for combined approaches with the mean time of 23 min for the frontal osteotomy procedure, including the time to fixate the frontal bone flap with plates and screws at the end of the procedure.

No major intraoperative complications occurred. Minor postoperative bleeding was observed in two cases (9%), both managed conservatively. In the two patients with postoperative bleeding, the events were self-limited and resolved without surgical intervention. No cerebrospinal fluid (CSF) leaks, orbital injuries, or infections were reported. Histopathologic examination confirmed all specimens as benign osteomas, either compact or mixed types.

The average follow-up after surgery was about 40.3 months (range 20–68 months). Regarding clinical outcomes, among the 21 symptomatic patients, complete resolution of symptoms was achieved in the majority of cases, whereas only 4 patients (19%) reported partial improvement. Headache was the only symptom that persisted in these patients, even if it occurred with reduced frequency and intensity. No other residual symptoms were observed at the latest follow-up.

No radiological or endoscopic evidence of residual or recurrent disease was observed during the follow-up period. No cases of mucocele formation were observed during the available follow-up period. Among the six patients treated with combined approaches, no clinically evident scars, forehead contour irregularities, or other aesthetic sequelae were observed at the latest follow-up. As no validated cosmetic assessment scale was administered, aesthetic outcomes were reported descriptively.

## Discussion

Paranasal sinus osteomas are slow-growing benign osseous tumors that remain asymptomatic in most cases. When symptoms occur, they are usually due to sinus outflow obstruction, mucocele formation, or orbital and intracranial compression.

From an anatomical perspective, the classification of paranasal sinus osteomas plays a central role in surgical planning. According to Prizio’s classification, lesions are stratified on coronal CT imaging based on their relationship with the lamina papyracea and the mid-orbital roof. Grade I osteomas are ≤ 3 cm and confined medially to the lamina papyracea, Grade II lesions are also ≤ 3 cm but extend laterally up to the mid-orbital plane, while Grade III osteomas exceed 3 cm or extend beyond the mid-orbital line, frequently involving the lateral frontal sinus or posterior Table [11].

More recent endoscopic series, including those reported by Castelnuovo and colleagues, have applied this anatomical framework in the context of contemporary endoscopic techniques, suggesting that surgical feasibility may vary according to factors beyond size and lateral extension alone, such as lesion attachment, sinus anatomy, and available instrumentation [12]. Regarding surgical indications, the literature consistently highlights that decisions should not be based only on symptomatology [17]. Although symptoms such as headache, nasal obstruction, recurrent sinusitis, orbital displacement, diplopia, or epiphora remain a major trigger for intervention, the literature emphasizes that surgical indications should incorporate radiological progression, anatomical risk factors, and long-term safety considerations.

Early studies by Savic and Djurić supported observation for small, stable frontal sinus osteomas that do not obstruct the frontal recess and show no growth over time [18]. However, surgery is recommended even in asymptomatic patients when the osteoma involves the ethmoid sinus, when the lesion occupies more than 50% of the frontal sinus, in case of measurable enlargement on sequential imaging, or involvement of nasofrontal duct, orbit, or anterior cranial fossa. More recent evidence, including the criteria proposed by Sofokleous, suggests that lesions growing > 1–2 mm/year or demonstrating intracranial or orbital extension need early resection due to the increased risk of complications and greater surgical difficulty if intervention is delayed [19]. Nowadays, the areas of greatest debate concern not the indication to surgery, but rather the choice of the most adequate surgical approach and procedure. While several authors support a purely endoscopic approach for small, medially located Grade I and II osteomas, they recommend combined or open techniques for Grade III lesions, in which far-lateral extension or posterior wall involvement limits safe endonasal access. This discrepancy in proposed strategies reflects variations in anatomical interpretation, surgical expertise, and technological capabilities among different centers [20]. 

Basing on these considerations, the present study aimed to report a series of osteomas in different stages, surgically treated at our institution, and to develop a decision-making flowchart that may assist surgeons in selecting the most appropriate surgical approach according to lesion grade and anatomical accessibility, as well as defining the specific procedures required for each category.

Unlike existing grading systems, the proposed flowchart incorporates additional anatomical and technological variables that directly influence surgical planning, including lesion attachment, frontal sinus table involvement, restoration of sinus drainage, intraoperative navigation, and patient-specific cutting guides. This approach provides a practical decision-making framework for contemporary management of frontal and frontoethmoidal osteomas.

Surgery is recommended for symptomatic osteomas, for lesions that demonstrate progressive growth, or for selected asymptomatic cases located in the frontal recess where early endoscopic removal may prevent future obstruction. Conversely, small and stable lesions in non-critical areas can be safely monitored with periodic imaging. Once surgery is indicated, the osteoma is classified anatomically. Type I lesions are managed with a purely endoscopic approach, while Type II lesions are treated endoscopically unless anatomical limitations require a combined technique. Type III osteomas, especially in case of lateral or posterior extension, require a combined endoscopic–external approach. Preservation of frontal sinus drainage was preferred whenever a patent and functional frontal outflow tract could be restored after tumor removal. Conversely, frontal sinus obliteration was reserved for selected Grade III lesions characterized by extensive sinus occupation, severe distortion of the frontal recess, or the need for extensive mucosal removal, which rendered restoration of physiological drainage unreliable. Given the potential risk of delayed mucocele formation after frontal sinus obliteration, long-term surveillance is advisable. Mucocele development has been associated mainly with residual sinus mucosa, incomplete obliteration or postoperative obstruction of the frontal recess, and scarring of the drainage pathway. Since this complication may occur several years or even decades after frontal sinus surgery, a prolonged clinical follow-up is warranted. In the present series, a minimum follow-up of 18 months was required, with postoperative CT evaluation at 12 months and additional imaging performed when clinically indicated. No cases of mucocele formation were observed during the available follow-up period. In these cases, our algorithm incorporates a patient-specific planning protocol: CT data are segmented to create a 3D model, optimal osteotomy lines are defined, and a custom-made cutting guide is fabricated. The combined use of intraoperative navigation, high-definition endoscopy, and 3D patient-specific tools is essential for safe and effective management of complex lesions [14, 15].

The proposed flowchart is not intended to replace existing classification systems, but rather to complement them by integrating clinical, radiological, anatomical, and technological findings into a practical decision-making tool for selecting the most appropriate surgical approach. In our series, the surgical strategy was fully consistent with the proposed algorithm. In the present series, the surgical procedure was tailored according to osteoma type. Type I lesions were managed endoscopically through uncinectomy, anterior ethmoidectomy, and Draf IIa frontal sinusotomy. Type II osteomas required an extended endoscopic approach consisting of uncinectomy, anterior and posterior ethmoidectomy, and Draf IIb frontal sinusotomy. Type III lesions were treated through a combined endoscopic–external approach, including uncinectomy, anterior and posterior ethmoidectomy, Draf IIb frontal sinusotomy, and external opening of the frontal sinus through elevation of the frontal bone bandeau. Frontal sinus obliteration was performed when restoration of physiological drainage was not feasible or when posterior table erosion was present. No cases deviated from this treatment pathway.

**Fig. 8:**
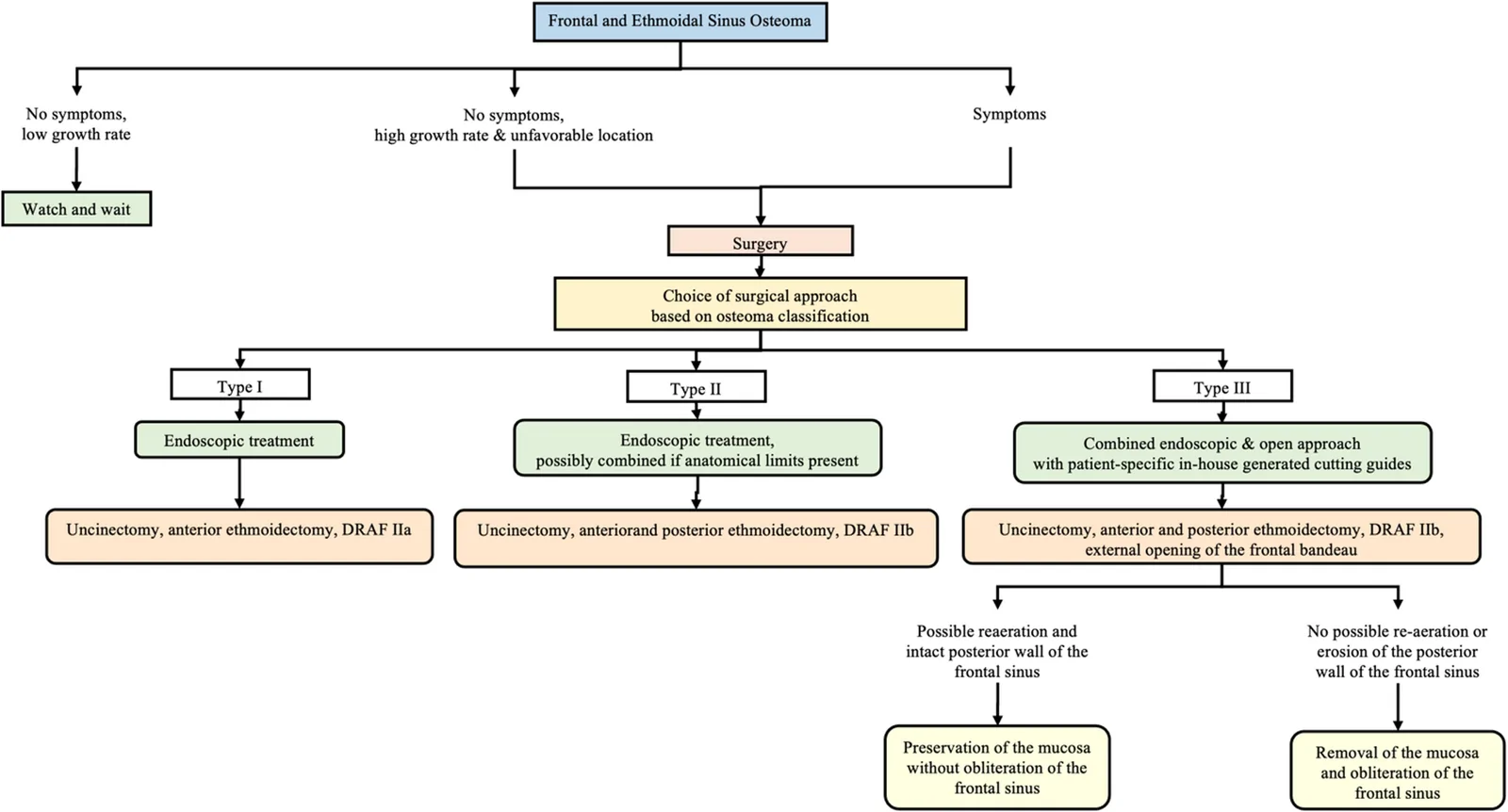
Proposed flowchart for the surgical management of frontoethmoidal osteomas. Type I: medially to the lamina papyracea. Type II: extending laterally up to the midpoint of the orbital roof. Type III: extension lateral to the mid-orbital plane into the lateral frontal sinus

The developed decision-making flowchart is summarized in Fig. 8.

The distribution of lesions in our population (68% frontal, 23% fronto-ethmoidal, 9% ethmoidal) aligns with large published series reporting frontal predominance ranging from 60% to 80% of cases [21]. The mean patient age of 51.5 years and slight male predominance also correspond to prior epidemiological data. Surgical indications in our group were therefore based on a combination of symptomatology, growth pattern, and anatomical accessibility [22]. 

Overall, 72.7% of patients underwent successful resection using a purely endoscopic endonasal technique, whereas 27.3% required a combined endoscopic–osteoplastic approach. This distribution reflects the broader shift toward minimally invasive strategies, supported by continuous technological advancements [23]. 

Our results compare favorably with the largest available series, which reported endoscopic resection rates between 50% and 75%, with recurrence rates ranging from 0% to 3%. The postoperative outcomes in this series, complete symptom resolution in 81.8% of patients (18/22) and partial improvement in 18.2% (4/22), demonstrate that, when appropriately selected, both endoscopic and combined approaches provide effective and safe management of paranasal sinus osteomas. Moreover, our operative times, blood loss, and hospital stays were comparable to those reported in recent meta-analyses, confirming the efficiency of this approach [24]. 

Our results validate the algorithm’s reliability and align with the outcomes reported in the largest series available in the literature, confirming that a tailored, technology-assisted strategy can optimize both surgical and functional outcomes in the surgical management of paranasal sinus osteomas.

The main limitations of this study include the retrospective design and single-institution setting, which may limit generalizability. The sample size (*n* = 22) restricts the statistical power for subgroup analysis, although the homogeneity of surgical management provides internal consistency. A limitation of this study is its retrospective design. Moreover, the absence of a detailed radiologic quantitative assessment, including volumetric or three-dimensional morphometric analysis, limits the ability to precisely evaluate tumor growth patterns, anatomical distortions, and the extent of postoperative cavity remodeling. Advanced 3D segmentation could have provided more objective metrics to support surgical decision-making and outcome interpretation [25, 26]. Potential selection bias cannot be excluded, as only patients who underwent surgical treatment were included; asymptomatic or conservatively managed osteomas were not analyzed, reducing the applicability of findings to the broader osteoma population. In addition, no control or comparison group was available for the combined approaches, considering that all procedures were performed using custom-made surgical cutting guides and intraoperative neuronavigation [27]. Finally, although all operations were performed by the same senior surgeon, ensuring technical consistency, this also implies an intrinsic learning curve. Over the study period, surgical expertise, familiarity with advanced endoscopic techniques, and integration of digital planning tools naturally evolved, potentially influencing operative performance and outcomes. Future multicenter prospective studies with standardized follow-up protocols and a control group are warranted to validate our algorithm and further assess cost-effectiveness and functional results.

## Conclusion

This study highlights the value of a structured, technology-assisted algorithm in guiding the surgical management of frontal and ethmoidal sinus osteomas. By integrating clinical presentation, anatomical assessment and modern surgical technologies, such as intraoperative navigation and patient-specific 3D cutting guides, the proposed model allows a safe, tailored, and reproducible approach to these challenging lesions. Endoscopic surgery remains the preferred option for most cases, while the combined technique ensures complete and controlled removal of complex or laterally extended osteomas. The favorable outcomes and absence of recurrences in our series support the reliability of this algorithm and its potential to improve both surgical precision and functional results in the management of paranasal sinus osteomas.

## References

[1] Sinha A, Jha D, Deka RC (2003) Osteoma of the paranasal sinuses. Indian J Otolaryngol Head Neck Surg 55(3):166–169 10.1007/BF02991944 PMC3451122 23119969

[2] Erdogan N, Demir U, Songu M, Ozenler NK, Uluç E, Dirim B (2009) A prospective study of paranasal sinus osteomas in 1,889 cases: Changing patterns of localization. Laryngoscope 119(12):2355–2359 10.1002/lary.20646 19780030

[3] Mafee MF, Tran BH, Chapa AR (2006) Imaging of rhinosinusitis and its complications: Plain film, CT, and MRI. Clin Rev Allergy Immunol 30(3):165–186 10.1385/CRIAI:30:3:165 16785588

[4] Smith ME, Calcaterra TC (1989) Frontal sinus osteoma. Ann Otol Rhinol Laryngol 98(11):896–900. 10.1177/000348948909801111 2817682

[5] Earwaker J (1993) Paranasal sinus osteomas: a review of 46 cases. Skeletal Radiol 22(6):417–423 10.1007/BF00538443 10.1007/BF00538443 8248815

[6] Lee DH, Jung SH, Yoon TM, Lee JK, Joo YE, Lim SC (2015) Characteristics of paranasal sinus osteoma and treatment outcomes. Acta Otolaryngol 135(6):602–607. 10.3109/00016489.2014.1003093 25719573

[7] Ataman M, Ayas K, Gürsel B (1993) Giant osteoma of the frontal sinus. Rhinology 31(4):185–187 8140386

[8] Minni A, Roncoroni L, Cialente F, Zoccali F, Colizza A, Placentino A, Ormellese G, Ralli M, de Vincentiis M, Dragonetti A (2023) Surgical Approach to Frontal and Ethmoid Sinus Osteomas: The Experience of 2 Metropolitan Italian Hospitals. Ear Nose Throat J 102(11):720–726 10.1177/01455613211016895 34176317

[9] Strek P, Zagólski O, Składzień J, Oleś K, Konior M, Hydzik-Sobocińska K, Głowacki R (2007) Endoscopic management of osteomas of the paranasal sinuses–own experience. Otolaryngol Pol 61(3):260–264. Polish 10.1016/S0030-6657(07)70423-7 17847778

[10] Chiu AG, Schipor I, Cohen NA, Kennedy DW, Palmer JN (2005) Surgical decisions in the management of frontal sinus osteomas. Am J Rhinol 19(2):191–197 15921220

[11] Prizio C, Achena A, Lorenzi A, Micolitti C, Accorona R, Pilolli F et al (2025) Optimizing surgical strategies for frontal sinus osteomas: a proposal for classification. Eur Arch Otorhinolaryngol 282(5):2419–2427 10.1007/s00405-025-09331-x 40155545

[12] Castelnuovo P, Valentini V, Giovannetti F, Bignami M, Cassoni A, Iannetti G (2008) Osteomas of the Maxillofacial District. J Craniofac Surg 19(6):1446–1452 10.1097/SCS.0b013e31818b417d 19098531

[13] Romano A, Troise S, Maffia F, Committeri U, Sani L, Sarcinella M, Arena A, Iaconetta G, Califano L, Dell’Aversana Orabona G (2023) Anteroposterior ethmoidectomy in the endoscopic reduction of medial orbital wall fractures: Does it really reduce stability? Appl Sci 13(1):98. 10.3390/app13010098

[14] Romano A, Troise S, Spinelli R, Abbate V, Dell’Aversana Orabona G (2025) Guided frontal sinus osteotomy: A pilot study of a digital protocol for in-house. Manuf Surg Cut Guides J Clin Med 14(9):3141. 10.3390/jcm14093141 PMC12072317 40364172

[15] Carraturo E, Germano C, Troise S, Spinelli R, Romano A, Giudice GL, Vaira LA, Piombino P (2025) In-house 3D printed surgical guide for frontal sinus osteotomy in traumatology: a technical note. J Stomatol Oral Maxillofac Surg 126(1):102023. 10.1016/j.jormas.2024.102023 39187038

[16] Bessen S, Gadkaree SK, Derakhshan A (2024) Use of piezoelectric instrumentation in craniofacial surgery. Curr Opin Otolaryngol Head Neck Surg 32(4):209–214. 10.1097/MOO.0000000000000986 38900192

[17] Mali S (2013) Paranasal sinus osteoma: review of literature. Oral Surg 7:1–6. 10.1111/ors.12066

[18] Savić DL, Djerić DR (1990) Indications for the surgical treatment of osteomas of the frontal and ethmoid sinuses. Clin Otolaryngol Allied Sci 15(5):397–404. x 10.1111/j.1365-2273.1990.tb00490.x 2282700

[19] Sofokleous V, Maragoudakis P, Kyrodimos E, Giotakis E (2021) Management of paranasal sinus osteomas: A comprehensive narrative review of the literature and an up-to-date grading system. Am J Otolaryngol 42(5):102644. 10.1016/j.amjoto.2020.102644 33799138

[20] Boysen M (1978) Osteomas of the paranasal sinuses. J Otolaryngol 7(4):366–370 691104

[21] Rampinelli V, Ferrari M, Dohin I, Vinciguerra A, Mattavelli D, Saccardo T, Testa G, Conti C, Frigerio C, Verillaud B, Ajasllari G, Gaudioso P, Cazzador D, Aga A, Taboni S, Daniele Arioso A, Neri L, Borsetto D, Wolf A, Rodriguez-Van Strahlen C, Turri-Zanoni M, Emanuelli E, Schreiber A, Battaglia P, Pagella F, Bignami M, Piazza C, Castelnuovo P, Nicolai P, Herman P (2025) Advanced paranasal sinuses osteomas: a retrospective multicentric analysis on surgical management outcomes and intra- and postsurgical complications. Rhinology 63(2):209–220. 10.4193/Rhin24.165 39831842

[22] Schick B, Steigerwald C, el Rahman el, Tahan A, Draf W (2001) The role of endonasal surgery in the management of frontoethmoidal osteomas. Rhinology 39(2):66–70 11486440

[23] Citardi MJ, Batra PS (2007) Intraoperative surgical navigation for endoscopic sinus surgery: rationale and indications. Curr Opin Otolaryngol Head Neck Surg 15(1):23–27. 10.1097/MOO.0b013e3280123130 17211179

[24] Crocetta FM, Farneti P, Sollini G, Castellucci A, Ghidini A, Spinosi MC, Fernandez IJ, Zoli M, Mazzatenta D, Pasquini E (2021) Endoscopic management of frontal sinus diseases after frontal craniotomy: a case series and review of the literature. Eur Arch Otorhinolaryngol 278(4):1035–1045. 10.1007/s00405-020-06335-7 32880737

[25] Abbate V, Committeri U, Troise S, Bonavolontà P, Vaira LA, Gabriele G, Biglioli F, Tarabbia F, Califano L, Dell’Aversana Orabona G (2023) Virtual surgical reduction in atrophic edentulous mandible fractures: a novel approach based on in-house digital workflow. Appl Sci 13:1474. 10.3390/app13031474

[26] Wauters LD, Miguel-Moragas JS, Mommaerts MY (2015) Classification of computer-aided design-computer-aided manufacturing applications for the reconstruction of cranio-maxillo-facial defects. J Craniofac Surg 26(8):2329–2333 10.1097/SCS.0000000000001845 26491918

[27] Palazzolo V, Tronchet A, Valsecchi S, Bellocchi S, Gervasoni C, Spotti S, Francesco AD, Colombo L, Riccio S, Ronchi P (2018) Removal of a frontal sinus osteoma and reconstruction by a custom-made implant with neuronavigation assistance. Craniomaxillofac Trauma Reconstr 11(4):305–313. 10.1055/s-0037-1607066 PMC6224279 30574275

